# Suppressing Thermal Noise to Sub-Millikelvin Level in a Single-Spin Quantum System Using Realtime Frequency Tracking

**DOI:** 10.3390/mi15070911

**Published:** 2024-07-13

**Authors:** Zhiyi Hu, Jingyan He, Runchuan Ye, Xue Lin, Feifei Zhou, Nanyang Xu

**Affiliations:** 1 School of Microelectronics, Hefei University of Technology, Hefei 230009, China; 2018111094@mail.hfut.edu.cn; 2 Institute of Quantum Sensing and College of Optical Science and Engineering, Zhejiang University, Hangzhou 310027, China; 2021111173@mail.hfut.edu.cn (J.H.); rcye@mail.hfut.edu.cn (R.Y.); xuel@mail.hfut.edu.cn (X.L.); 3 College of Metrology Measurement and Instrument, China Jiliang University, Hangzhou 310018, China

**Keywords:** NV center, thermal noise suppression, spin manipulation, realtime frequency tracking, feedback control

## Abstract

A single nitrogen-vacancy (NV) center in a diamond can be used as a nanoscale sensor for magnetic field, electric field or nuclear spins. Due to its low photon detection efficiency, such sensing processes often take a long time, suffering from an electron spin resonance (ESR) frequency fluctuation induced by the time-varying thermal perturbations noise. Thus, suppressing the thermal noise is the fundamental way to enhance single-sensor performance, which is typically achieved by utilizing a thermal control protocol with a complicated and highly costly apparatus if a millikelvin-level stabilization is required. Here, we analyze the real-time thermal drift and utilize an active way to alternately track the single-spin ESR frequency drift in the experiment. Using this method, we achieve a temperature stabilization effect equivalent to sub-millikelvin (0.8 mK) level with no extra environmental thermal control, and the spin-state readout contrast is significantly improved in long-lasting experiments. This method holds broad applicability for NV-based single-spin experiments and harbors the potential for prospective expansion into diverse nanoscale quantum sensing domains.

## 1. Introduction

A single nitrogen-vacancy (NV) center [1] in diamonds has emerged as an excellent multi-functional sensor to detect magnetic fields [2,3,4,5,6], electric fields [7,8,9,10], temperature [11,12,13,14], strain [15,16], or other spins [17,18,19] with high sensitivity and nanoscale spatial resolution. The single electron spin with nearby nuclear spins in the NV center also form a hybrid system that has been used as a quantum register [20,21,22] for computation [23,24] and simulation [25,26] tasks, or interacting nodes of the quantum network [27,28,29,30]. However, such experimental processes often have a large time cost due to the low photon collection efficiency of single NV, which restricts spin manipulation precision and read-out fidelity because of the thermally induced electron spin resonance (ESR) frequency drift, especially under the high-field conditions associated with an obvious magnetic field gradient [31]. Thus, mitigating the thermal noise is a crucial and direct way to improve the spin control accuracy and accomplish the reliable spin-based quantum sensing.

To address this issue, various methods have been developed and implemented in various experiments, including dynamical decoupling sequences [32,33,34,35] and increasing the excitation microwave power [36]. However, these methods typically necessitate precise control conditions and strong microwave power, which limits their potential applications. For instance, certain biological sensing experiments have strict requirements on the excitation microwave power [37]. A more general approach is to apply an environmental temperature control apparatus in experiments to stabilize the local temperature within a controllable range. But the assistance of a complicate apparatus is needed if high-precision stabilization is required, for example, beyond the mK level [38]. Moreover, the passive temperature control devices cannot cancel out the significant in situ heating of the NV center generated by the radiated microwave (MW) and pumping laser for spin manipulations [39]. There is also another alternative protocol to measure the thermally induced ESR frequency drift using optically detected magnetic resonance (ODMR) measurement and compensate the drift accordingly. Limited by the finite photon rates of single NV, generally this ODMR feedback method can only track the thermal drifts in a frequency range of mHz level.

Recently, various methods were developed to rapidly track the NV ESR frequency changes in real time. For example, a four-point detection protocol was designed for magnetic imaging or temperature measurement using NV single-spin or small ensembles [40]. Another fast ESR tracking method is the single-spin lock-in amplifier (LIA) detection technique [41,42] utilizing a frequency-modulated MW. With these techniques, the in situ thermal perturbations of NV center can be well characterized, referring to the temperature dependence of ESR frequencies.

In this paper, we utilize a real-time frequency tracking method based on LIA technology to analyze the thermally induced ESR frequency drift of single spin. By employing this method in experiments, we can achieve real-time resonance frequency tracking and update the experimental settings, actively counteracting thermal noise. We demonstrate the use of this method in a long-lasting Rabi experiment, observing a significant improvement in the readout contrast of the spin state. We also systematically evaluate the thermal stabilization performance and robustness of our method with different NV centers by studying the Rabi contrast behaviors under different experimental settings and predicting its equivalent thermal stabilization precision referring to the fitting results of conventional schemes. Assisted by the real-time frequency tracking method, the Rabi contrast reaches the expectation which can be achieved under a passive temperature control precision of 0.8 mK. Our results provide a promising robust control protocol for NV-based single-spin experiments and have the potential for prospective expansion into diverse nanoscale quantum sensing applications.

## 2. Experiment Setup

**Figure 1:**
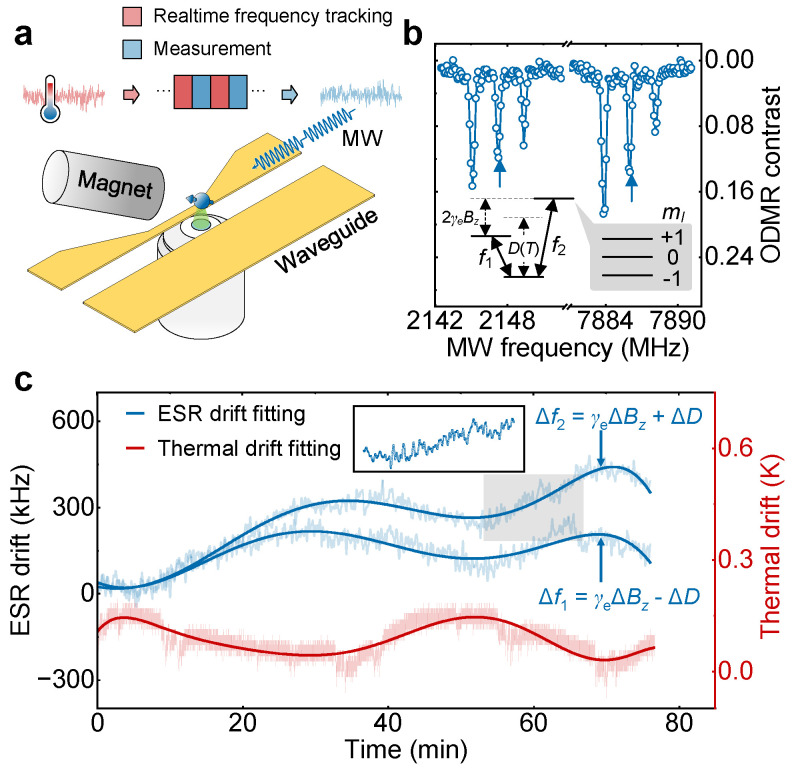
(Color online) (**a**) Schematic of the real-time frequency tracking scheme for thermal drift suppression. The red and blue blocks denote the real-time frequency tracking process and measurement sequence, respectively. (**b**) Typical ODMR spectrum of the NV center under an axial magnetic field $B_{z}$ of 1800 Gs. The resonances of |0〉↔|±1〉 are split into six dips due to the hyperfine interaction of ^14^N nucleus and can be well distinguished by reducing MW power. The ESR frequencies $f_{1}$ and $f_{2}$, as indicated using blue arrows, are mainly determined by axial magnetic field $B_{z}$ and environmental temperature *T*. (**c**) Real-time ESR frequency drifts of the NV center detected once per second and simultaneous temperature changes recorded by a thermometer with an interval of 600 μs. The inset is an enlarged version of the drawing of the gray-filled area.

The NV center consists of a vacancy with a neighboring nitrogen atom and its negatively charged state NV^−^ forms a spin triplet in the orbital ground state. The spin states of NV center can be manipulated by resonant MW and optically read out by a 532 nm laser. Here, we conduct the NV experiments based on a home-built ODMR setup, where both the permanent magnet and the diamond are placed into a temperature-stabilized container. The MW radiation for spin manipulation is applied by using a coplanar waveguide, as shown in Figure 1a. The spin Hamiltonian of NV center reads

(1) $$H=D\left(T\right)S_{z}^{2}-\gamma_{e}B_{z}S_{z}+H_{hf},$$

where D(T) is the zero-splitting parameter with a temperature dependence of −74.2 kHz/K [43], $S_{z}$ is the electron-spin operator, $\gamma_{e}B_{z}$ represents the Zeeman splitting which separates the degenerate |±1〉 states, and $H_{hf}$ denotes the hyperfine interaction contributed by the nearby nuclear spins. From the above spin Hamiltonian analysis, a change in external axial magnetic field of 1 Gs induces a resonance frequency shift of 2.8 MHz, whereas a temperature variation of 1 K leads to an approximate 74 kHz alteration in resonance frequency. As a consequence, we can find that the ESR frequency is mainly dominated by the external axial magnetic field $B_{z}$ and environmental temperature *T*.

In NV-based quantum sensing, ODMR spectroscopy is a fundamental and widely used method. The ESR frequencies are detected by sweeping the MW frequencies to obtain an ODMR spectrum and fitting the spectral dips, as shown in Figure 1b. However, ESR frequency drifts often occur during the experimental duration because of the temperature fluctuation, which not only changes the *D* parameter, but introduces the perturbations of the in situ magnetic field ($B_{z}$) of the NV center because of the thermally induced mechanical variations and deformations of the permanent magnet. Conventionally, there are two ways to mitigate the impact of temperature fluctuations. First, the commonly used approach in experiments is to frequently calibrate the ESR frequency and update the MW settings, typically at an interval of several hundred seconds. Besides, another hardware-based scheme is to place the sample and the permanent magnet into a high-precision passive thermal control container to stabilize the temperature.

In this work, we focus on the former method and utilize the real-time frequency tracking instead to accelerate the ESR measurements. Our device scans only a narrow range of MW frequencies, allowing us to acquire the time-varying drifts of bilateral ESR frequencies $f_{1}$ and $f_{2}$ within an interval of one second, as shown in Figure 1c. Specifically, the real-time frequency tracking method imposes a sine wave modulation with a frequency of 1 Hz and a depth of 80 kHz on a fixed-frequency microwave, thereby inducing a periodic disturbance in the output microwave. This modulation is phase-locked with an external oscillation reference. When the electron spin resonates with the MW field, this periodic disturbance induces synchronous modulation in the fluorescence emitted by NV centers. A purpose-built mixed-signal data acquisition (DAQ) system concurrently captures both the analog reference signal and the fluorescence counts from the avalanche photodiode (APD). In the presence of a minor detuning δ in the MW frequency, the in-phase amplitude of the modulated fluorescence correlates approximately with the first derivative of the ODMR signal. Consequently, by maintaining a fixed and stable MW frequency, the resonance frequency drift can be inferred by extracting this amplitude from the detection, thus allowing precise determination of the current resonance frequency. Thanks to the real-time frequency tracking detection, the higher-frequency component of ESR frequency drifts can be observed; see the inset of Figure 1c. The ESR frequencies slowly change within a long time duration, agreeing well with the simultaneous trace of thermal drift. Referring to the Equation (1), the drift of $\gamma_{e}B_{z}$ (*D*) corresponds to the sum (difference) of $\Delta f_{1}$ and $\Delta f_{2}$.

## 3. Results

**Figure 2:**
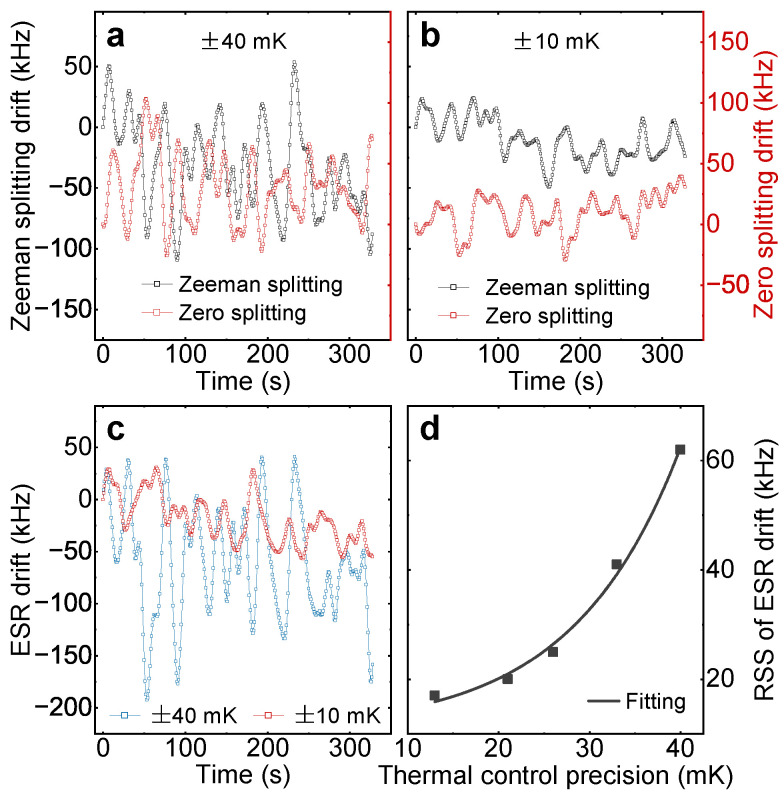
(Color online) (**a**,**b**) Real-time drifts of the Zeeman splitting $\gamma_{e}B_{z}$ and zero splitting *D* referring to the ESR frequency ($f_{1}\&f_{2}$) drifts of NV-1 center with different thermal stabilization precision at 1800 Gs. (**c**) Real-time drift of $f_{1}$ corresponding to (**a**,**b**). The RSS value of $f_{1}$ drift under ±40 mK (±10 mK) thermal condition is 56 kHz (20 kHz). (**d**) RSS value behaviors of $f_{1}$ drift as a function of thermal stabilization precision. The results are well fitted by the exponential growth function.

We then systematically study the behaviors of the $\gamma_{e}B_{z}$ and *D* within different thermal dynamical ranges, respectively, by employing a passive precision-controllable temperature stabilization system and tracking the ESR frequencies. For the comparison, we use the residual sum of squares (RSS) of ESR frequency $f_{1}$ drift to quantify the thermal impact. We use a high-sensitivity thermometer to continuously monitor temperature changes inside the temperature-controlled box, with a sampling interval of 600 μs. The RSS of the real-time temperature data collected over several hours represents the current temperature control precision. In experiments, different levels of temperature control precision can be achieved by finely adjusting the parameters of the proportional–integral–derivative (PID) feedback temperature control. The tracking results with a precision of ±40 mK are depicted in Figure 2a. After improving the precision up to approximately ±10 mK, both the fluctuation amplitudes of $\gamma_{e}B_{z}$ and *D* obviously decrease, as shown in Figure 2b. The corresponding $f_{1}$ drifts are illustrated in Figure 2c, where the RSS value decreases by nearly one-third after improving the precision. We also study the RSS value behaviors as a function of thermal stabilization precision shown in Figure 2d. One can find that the temperature stabilization effectively suppresses the ESR frequency drift. We performed the Fourier transform on the oscillations signal and found that its frequency components are mainly concentrated around 50 mHz. In most experiments, we typically initiated ODMR measurement every five minutes for resonant frequency calibration, corresponding to a sampling rate of approximately 3 mHz. This makes the ODMR insufficient to suppress the thermally induced noise at the NV center. In contrast, the lock-in method, with a feedback speed of 400 mHz, can effectively suppress the impact of thermally induced noise on the NV center.

**Figure 3:**
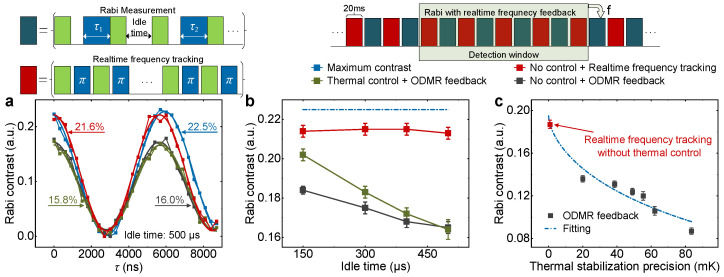
(Color online) (**a**) Rabi experiments of NV-1 center with different control schemes at 1800 Gs. Due to the application of weak microwave power, the Rabi period is 5600 ns. In the Rabi sequence, green-filled blocks represent the spin polarization and readout using 532 nm laser pulses. The blue-filled blocks are the MW pulses with a duration of $\tau_{1(2)}$. The idle time for achieving maximum Rabi contrast is set to 1 μs, and the other times are set to 500 μs. (**b**) Rabi contrast behaviors of NV-1 center as a function of idle time with different control schemes. The idle times are set to 150 μs, 300 μs, 400 μs, and 500 μs, respectively. (**c**) Rabi contrast behaviors of NV-2 center as a function of thermal stabilization precision. For the NV-2 center, the achieved maximum Rabi contrast with an idle time of 1 μs is 19.6%. The black dots represent experimental results with an idle time of 300 μs using ODMR feedback. When employing the real-time frequency tracking protocol without thermal control, Rabi contrast is significantly improved up to 18.7%, which corresponds to an equivalent precision of 0.8 mK (red dot).

Following the above analysis, we investigate the thermal stabilization efforts on the spin manipulation as well. The Rabi oscillation serves as the foundation for a wide range of sensing experiments, and can directly evaluate the spin control precision. For the comprehensive comparisons, here, we design and conduct four groups of Rabi experiments with different control schemes on an NV center (NV-1). First, we calibrate the expected maximum Rabi contrast of the NV-1 center by setting the idle time between two neighboring measurement points as 1 μs to minimize the thermal impact. As a result, a maximum Rabi contrast of 22.5% is measured, as shown in Figure 3a. Second, we utilize the ODMR measurement to periodically update the ESR frequency and feed it back to the MW system without thermal control. To imitate the thermal impact on the long-lasting experiments, we increase the idle time up to 500 μs, thereby extending the entire experimental duration and obtaining a reduced Rabi contrast of 16.0%. Third, based on the second group, we introduce a ±10 mK passive thermal stabilization to assist the Rabi measurement. However, the corresponding contrast differs from the second group by only 15.8%, which means that this precision cannot satisfy the requirement for the long-lasting experiment.

Finally, we abandon the passive thermal stabilization and utilize our real-time frequency tracking method instead to rapidly track and update the ESR frequency drifts. As indicated in Figure 3a, frequency feedback and Rabi measurement are alternately executed by an interval of 20 ms. Following the accumulation of adequate signals throughout a full detection window, the present resonance frequency is provided as feedback to the microwave system. Due to limitations imposed by the photon detection rate, the feedback speed is currently around 2.5 s, with a 10 s delay observed between the detected resonance frequency and the true frequency (equivalent to half the width of the detection window). In this time, the Rabi contrast is obviously improved up to 21.6%, approaching the maximum contrast even without passive thermal control. This enhancement presents the excellent performance of our real-time frequency tracking method on the thermal perturbation suppression.

To comprehensively demonstrate the performance of our method during the long-lasting experiments, we vary the idle time of the Rabi sequence and compare the efforts with conventional schemes. Figure 3b shows the Rabi contrast behaviors as a function of idle time. With the increase in the idle time, the thermal stabilization efforts of conventional schemes decrease, while our real-time frequency tracking scheme always have a robust suppression effect on the thermal impact.

We investigate the Rabi contrast behaviors as a function of passive thermal stabilization precision on another NV center (NV-2) to confirm the robustness of our method and evaluate its equivalent performance, as depicted in Figure 3c. The maximum Rabi contrast of the NV-2 center is predetermined to be 19.6%. Although it utilizes passive temperature control and ODMR feedback schemes, the Rabi contrast is still not good enough. With the aid of real-time frequency tracking, Rabi contrast is significantly protected and reaches 18.7% close to the maximum even without extra passive thermal control. Since the Rabi contrast will disappear (approaching 0) when the thermal perturbation is too strong, here, we employ an exponential decay model to fit the data in Figure 3c. Referring to the fitting, the equivalent thermal stabilization precision of the real-time frequency tracking method is predicted to be 0.8 mK.

## 4. Discussion

Here, we demonstrate a real-time frequency tracking method to track the ESR frequencies of the NV center in real-time quantum sensing experiments and actively resist the thermal noise. The real-time frequency tracking method focuses on local changes in the resonance frequency and operates synchronously with the experiment. Consequently, the rapid resonance frequency update rate ensures the protocol’s ability to suppress thermal noise. The suppression effect of this method is equivalent to applying an active closed-loop thermal stabilization instrument with a precision of 0.8 mK. Compared with the traditional temperature control protocols, our scheme requires no complicated setup and can also compensate the in situ laser and microwave heating impact, which cannot be realized by traditional apparatus. Currently, the performance of our scheme is mainly limited by the detection window width, which induces the feedback time latency. To further enhance the protocol performance, we can use emitting-enhanced techniques such as a solid immersion lens [44,45] and pillar to improve the photon collection efficiency [46,47] or use the ensemble NV centers [48] thereby reducing the signal detection time. Also, combining machine learning to compensate the detection latency is another promising choice [49]. Then, the frequency tracking speed is increased, which is advantageous for handling higher-frequency noise.

Our method can be applied to more general spin-based quantum sensing fields, for example, the low excitation power nuclear spin probing experiments. For experiments with a significant time cost under high magnetic fields, our method can substitute traditional temperature control apparatus and achieve better suppression of thermal noise [50]. Moreover it can enhance spin manipulation accuracy and enables reduced microwave power usage in high-field environments, thereby benefiting biological experiments. The method can also find applications in other quantum spin systems such as color centers in silicon carbide [51,52]. Due to the low signal-to-noise ratio of silicon carbide, a significant time investment is required to accumulate the necessary signals. Real-time frequency tracking enables better noise suppression and improves the signal contrast. This approach is also applicable to other quantum materials, such as emerging two-dimensional and organic materials. Notable examples include spin defects in hexagonal boron nitride [53,54,55] and pentacene in the lattice of *p*-terphenyl [56].

## Data Availability

Data are contained within the article.
